# HCV Core Protein Induces Chemokine CCL2 and CXCL10 Expression Through NF-κB Signaling Pathway in Macrophages

**DOI:** 10.3389/fimmu.2021.654998

**Published:** 2021-08-31

**Authors:** Xiaotian Song, Xue Gao, Yadong Wang, Rameez Raja, Yaoyu Zhang, Shulin Yang, Miao Li, Zhiyan Yao, Lin Wei

**Affiliations:** ^1^Department of Immunology, Hebei Medical University, Shijiazhuang, China; ^2^Key Laboratory of Immune Mechanism and Intervention on Serious Disease in Hebei Province, Shijiazhuang, China; ^3^Department of Infectious Diseases, The Third Hospital of Hebei Medical University, Shijiazhuang, China; ^4^Department of Inflammation and Immunity, Lerner Research Institute, Cleveland Clinic, Cleveland, OH, United States

**Keywords:** HCV core protein, macrophages, chemokines, CCL2, CXCL10

## Abstract

HCV core protein is the first structural protein synthesized during hepatitis C virus (HCV) infection and replication. It is released from virus infected liver cells and mediates multiple functions to affect host cell response. The innate immune response is the first line of defense against viral infection. After HCV infection, Kupffer cells (KCs) which are liver macrophages play an important role in host innate immune response. Kupffer cells act as phagocytes and release different cytokines and chemokines to counter viral infection and regulate inflammation and fibrosis in liver. Earlier, we have demonstrated that HCV core protein interacts with gC1qR and activates MAPK, NF-κB and PI3K/AKT pathways in macrophages. In this study, we explored the effect of HCV core protein on CCL2 and CXCL10 expression in macrophages and the signaling pathways involved. Upon silencing of gC1qR, we observed a significant decrease expression of CCL2 and CXCL10 in macrophages in the presence of HCV core protein. Inhibiting NF-κB pathway, but not P38, JNK, ERK and AKT pathways greatly reduced the expression of CCL2 and CXCL10. Therefore, our results indicate that interaction of HCV core protein with gC1qR could induce CCL2 and CXCL10 secretion in macrophages *via* NF-κB signaling pathway. These findings may shed light on the understanding of how leukocytes migrate into the liver and exaggerate host-derived immune responses and may provide novel therapeutic targets in HCV chronic inflammation.

## Introduction

Hepatitis C virus (HCV) imposes a significant burden on global healthcare (1, 2). It tends to cause chronic liver disease and often leads to fibrosis/cirrhosis, liver failure and hepatocellular carcinoma (HCC) (3–6). HCV is a single-stranded positive-sense RNA virus. Its genome encodes a 3000 residue polyprotein which is cleaved by host and viral proteases into three structural proteins (core, E1 and E2 protein) and seven nonstructural proteins (p7, NS2, NS3, NS4A, NS4B, NS5A, and NS5B) (7–11). HCV core protein (21kDa) is the first protein to be synthesized upon virus infection and takes part in the various activities of virus life cycle and assembling of virus particles (12). It is released from virus infected liver cells and exhibits multiple functions to affect host immunity by interacting with immunocytes (13–15). Tu et al. have reported that HCV core protein manipulates human blood-derived dendritic cell development and promotes Th17 differentiation (16). Zhai et al. have demonstrated that HCV core protein triggers CD4^+^CD25^+^Treg activation and expansion to inhibit host immune responses which may be involved in the viral persistence in HCV infected patients (17). HCV core protein has been shown to down-regulate suppressor of cytokine signalling-1 (SOCS-1) and up-regulate signal transducer and activator of transcription-1 (STAT-1) phosphorylation in B cells (18). HCV core protein acts as NOD-like receptor family pyrin domain-containing 3 (NLRP3) inflammasome agonist that drives inflammasome assembly leading to the production/release of bioactive IL-1β from macrophages (19). Galli et al. have reported that HCV core protein can be tested in serum for diagnosis of HCV infection and has the same sensitivity as HCV-RNA testing for screening and monitoring the process of infection (20). In this study, we focused on HCV core protein and macrophages interaction, especially on how HCV core protein affected macrophages chemokine secretion.

Kupffer cells (KCs) are macrophages that are found in the sinusoids of the liver. These cells serve as sentinels for maintaining liver homeostasis. KCs are a crucial part of the innate immune system and form the first immune barrier of the portal system. Liver macrophages are very plastic and mediate different functions depending on the signals of hepatic microenvironment (21). Wang et al. have reported that HCV dsRNA-activated macrophages have the ability to suppress HCV replication in hepatocytes by producing IFN-α/β (22). However, Ahmed et al. have demonstrated that the differentiation of M1 macrophages were impaired from HCV-infected individuals, which have significantly decreased IFN-γ expression in CD8^+^T cells (23). Ohtsuki et al. have suggested that M2 macrophages have the potential to produce proinflammatory cytokines in the liver of HCV transgenic mice and are responsible for liver inflammation (24). Although data from different labs suggest that macrophages play an important role in the process of HCV infection, the underlying mechanism of macrophages in HCV chronic progression remains unclear.

gC1qR is a multifunctional cellular protein expressed in a variety of tissues and cell types, including endothelial cells, dendritic cells, lymphocytes, and platelets (25–29). gC1qR mediates a myriad of biological responses including infection, inflammation, and immune regulation (30). As reported in the literature, gC1qR is gradually being recognized as an important pathogen recognition receptor (PRR). A wide range of bacterial and viral ligands are able to exploit gC1qR to either suppress the host’s immune response and thus enhance their survival, or to gain access into cells to initiate disease (31). For example, a direct interaction between InlB(invasion protein of *Listeria monocytogenes)* and gC1qR could promote the entry of *Listeria monocytogenes* into mammalian cells (32). The 3S motif of HIV-1 gp41 binding to gC1qR on CD4^+^T cells mediated NKp44L surface expression on CD4^+^T cells, which renders CD4^+^T cells sensitive to autologous NK lysis (33). In this study, we confirmed the interaction of HCV core protein with gC1qR in macrophages and its consequences on inflammatory response.

Sustained inflammation is an important factor for the progression of chronic liver diseases. NF-κB is known for its crucial role during immune and inflammatory responses. Activation of NF-κB pathway promoting the expression of pro-inflammatory cytokines, adhesion molecules, matrix degrading enzymes (MMPs) and so on which play important roles in initiation and perpetuation of chronic inflammation (34). Chemokines belong to cytokines that govern leukocyte trafficking and positioning into targeted organs (35). They help to shape inflammatory microenvironment and hold a major influence on the outcome of infection. Our previous study demonstrated that HCV core protein could trigger the activation of NF-κB pathway in macrophages (36). In addition, we examined the inflammation-associated gene expression in macrophages after treatment with HCV core protein by PCR Array analysis. A series of chemokines were increased, we focused on two chemokines CCL2 and CXCL10 which upregulated significantly. CCL2 promotes macrophage and T cells accumulation and inflammation while CXCL10 regulates the immune responses of T cells and natural killer cells (37). Here, we explored the mechanism for CCL2 and CXCL10 expression in macrophage in the presence of HCV core protein and highlight the function of CCL2 and CXCL10 in chronic inflammation. Our study provides a new perspective to understand the role of macrophages in chronic HCV inflammation.

## Materials and Methods

### Chemicals and Reagents

HCV core protein was expressed and purified in our lab as described earlier (36). It was prepared as lyophilized protein and was dissolved by PBS for use. IKKβ inhibitor (IMD 0354), Akt inhibitor (MK 2206 2HCl), JNK inhibitor (SP600125), P38 inhibitor (SB203580) and ERK inhibitor (PD98059) were obtained from Selleckchem. Phorbol 12-myristate 13-acetate (PMA, P1585) was obtained from Sigma. The antibodies anti-phospho-JNK (4668), anti-phospho-P38 (4511), anti-phospho-ERK (4370), anti-phospho-NF-κB p65 (3033), anti-NF-κB p65 (4764), anti-phospho-AKT (4060) were purchased from Cell Signaling Technology. Anti-JNK (BS3630), anti-P38 (BS3567), anti-ERK (BS1112), anti-AKT (MB0052), anti-GAPDH (AP0063) were purchased from Bioworld Technology. Anti-FLAG (M185-3L) was obtained from MBL and anti-HCV Core protein (SC-57800) was obtained from Santa Cruz. Secondary antibodies HRP-labeled goat-anti-mouse (ASS1007) and HRP-labeled goat-anti-rabbit (ASS1009) were purchased from Abgent.

### Plasmids

Promoter reporter plasmid of CCL2, corresponding to the sequence from -2000 to 0 (relative to the transcriptional start site) of the 5’-flanking region of the human CCL2 gene was generated from human genomic DNA using forward 5’-cctgagctcgctagcctcgagGAGAGAGGTTTCCCCTGATATGAG-3’, and reverse 5’-cagtaccggattgccaagcttAGGAGGAGGGATCTTCCATGAG-3’ primers. Promoter reporter plasmid of CXCL10, corresponding to the sequence from -2000 to 0 (relative to the transcriptional start site) of the 5’-flanking region of the human CXCL10 gene was generated from human genomic DNA using forward 5’-cctgagctcgctagcctcgagCACAGTTAATGTAATACAATGTTTAGTAAAAATC-3’, and reverse 5’-cagtaccggattgccaagcttAGAAAACGTGGGGCTAGTGTGC-3’ primers. The amplified product was gel purified and ligated into pGL4.10 Basic vector using Homologous recombination system (Vazyme). gC1qR expression plasmid (pCDNA3.1-SBP-Flag-gC1qR) and HCV core protein expression plasmid (pcDNA3.1-HCV core) plasmids were generated by Homologous recombination system (Vazyme). All constructs were sequenced from Sangon Biotech. Plasmid pmCherry-HA-p65 (human) was purchased from Miaolingbio. pGL4.10 Basic vector and pRL-TK plasmids were obtained from Promega. Plasmid lentiCRISPRv2-sgRNA-gC1qR, psPAX2 and pMD2.G were obtained from Bio-Change Technology.

### Cell Lines and Cell Culture

The human monocytic leukemia cell line THP-1 from the resource center of Peking Union Medical College Hospital (Beijing, China) and HEK 293T cells from Shanghai stem cell bank (Shanghai, China) were maintained in RPMI 1640 medium (Gibco) supplemented with 100 U/ml penicillin and 100 μg/ml streptomycin (Solarbio), 10 mM HEPES (Biosharp), and 10% fetal bovine serum (Gibco) at 37°C with 5% CO_2_ in a humidified atmosphere. Prior to experiments, human monocytes (THP-1) derived macrophages (MΦ-THP-1) were generated by phorbol 12-myristate 13-acetate (PMA, 20 ng/mL) treatment for 48 h followed by resting cells for 12 h according to the methods in references (38–40). The mouse macrophage cell line RAW 264.7 was cultured in Dulbecco’s modified Eagle’s medium (Gibco) supplemented with 100 U/ml penicillin and 100 μg/ml streptomycin, 10 mM HEPES, and 10% fetal bovine serum at 37°C with 5% CO_2_ in a humidified atmosphere. All cell lines were cultured within 10 passages prior to use. gC1qR knock-down cell line (THP-1-sh-gC1qR) and control cell line (THP-1-sh-luciferase) were prepared as previously described (36). gC1qR-knockout cell line (THP-1-KO-gC1qR) were generated as described in *Materials and Methods* below.

### Mouse Kupffer Cell Isolation and Purification

Mouse Kupffer cell isolation and purification were performed according to the method in the references (41, 42). The BALB/c mouse liver was removed aseptically and shred to small pieces followed by adding 5 mL of type IV collagenase (0.5mg/ml) to the petri dish and mixing it. It was kept on a shaking table at 37°C for full digestion for 15 min. The homogenate was filtered through a 100 µm cell strainer and subjected to centrifugation at 50 × g for 2 min at 4°C. The Parenchymal cells (hepatocytes) were in the pellet fraction and non-parenchymal cells (including Kupffer cells) in the supernatant fraction. The supernatant was transferred in a clean tube and centrifuged at 50 × g for 2 min. This step was repeated three times using RPMI 1640 medium followed by centrifugation at 1,350 × g for 15 min to pellet non-parenchymal cells. The supernatant was discarded and the pellet was resuspended in 10 ml RPMI 1640 medium. 700 mL/L Percoll solution, 300 mL/L Percoll solution and 3 mL of cell suspension was added slowly along the inner wall of 15 mL centrifuge tube to ensure clear boundaries of each layer. This was followed by centrifugation at 1,500 × g at 4°C for 20 min and collection of the intermediate cell layer between 700 mL/L and 300 mL/L solution and transferring into another centrifuge tube containing 10 ml of RPMI 1640 medium and centrifuged at 500 × g for 3 min. Pellet cells should be washed two times using RPMI 1640 medium by centrifugation at 500 × g for 3 min at 4°C followed by resuspending cells in 5-10 ml of pre-warmed RPMI 1640 Medium containing 10% fetal bovine serum. Cells were seeded in 12-well plates at a density of 1 × 10^6^ cells/well and incubated for 72 h. The adherent cells on the plates are KC cells.

### Monolayer Cultures of Mouse Peritoneal Macrophages

Mouse peritoneal macrophages (MPM) were harvested from the peritoneal cavities of BALB/c mice. They were centrifuged at 800 × *g* for 10 min and suspended in RPMI 1640 medium containing 10% fetal bovine serum. The macrophage suspension was applied to 12-well tissue culture plates with 1 × 10^6^ cells/well. The suspensions were incubated at 37°C for 4 h, washed thoroughly to remove non-adherent cells and further incubated overnight. The adherent cells on the plates are mouse peritoneal macrophages.

### CRISPR-Cas9 Gene-Editing Approach to Generate gC1qR Knockout Cells

Lentiviral particles were generated by transfection HEK293T cells with lentiCRISPRv2-sgRNA-gC1qR, psPAX2 and pMD2.G at a ratio of 4:3:1, respectively. Viral supernatants were collected 48 h after transfection. THP-1 cells were infected with lentivirus for 24 h and then treated with Puromycin for selection (1μg/mL, Biosharp). After Puromycin selection, cells were seeded as single colonies in 96-well plates. After 2 weeks, clones were selected based on western blotting with gC1qR antibody. In addition, genomic DNA was extracted from the cell lines arising from single clones and PCR analysis were performed to amplify targeted loci. Agarose gel electrophoresis was used to confirm the correct size of PCR products. PCR products were then cloned into the ZTOPO-Blunt/TA vector and transformed into DH5α competent cells. Plasmid DNA was isolated from multiple colonies of each transformation and sequenced to ensure frameshift mutations in the targeted region. The sequence information for sgRNAs used for gC1qR knockout (KO) cell generation is as follows:

gC1qR sgRNA1: CAGGAGCTGCCGGAAAGGCG

gC1qR sgRNA2: GTGCTGGGCTCCTCCGTCGC

### PCR Array Analysis

Differential expression of human inflammatory genes was analyzed using the RT² Profiler™ PCR Array obtained from Kangchen Genechip, Shanghai, China. MΦ-THP-1 cells were plated at a density of 1.2 × 10^7^ cells/10 cm dish and treated with HCV core protein (10 μg/mL) for 8 h (MΦ-THP-1 treated with PBS as control group). These cells were collected and RNA was extracted according to manufacturer’s protocol and converted to cDNA using the RT^2^ First Strand Kit. The template DNA was used for qPCR analysis using SYBR Green qPCR Master Mix. The threshold cycle (Ct) values for all the genes on each PCR Array were calculated using the instrument specific software and the fold-changes in gene expression were calculated using the ΔΔCT method. Filter criteria of differential expression mRNA was that fold change was greater than 2 and p value is less than 0.05.

### Real-Time Quantitative PCR

MΦ-THP-1 cells were plated at a density of 2 × 10^6^ cell/well in a 6-well plate, and treated with HCV core protein (10 μg/mL) for 8 h. Total RNA was extracted from cells using RNAiso Plus Reagent (TaKaRa) according to the manufacturer’s protocol, and then examined by detecting A260/A280 as well as by agarose gel electrophoresis. The RNA was reverse transcribed to cDNA using the Prime Script RT reagent kit with genomic DNA (gDNA) Eraser (TaKaRa). Quantitative real-time PCR (qPCR) was performed with cDNA templates and the Power Up SYBR green master mix (Applied Biosystems) and analyzed using the ABI prism 7500 Sequence detection system (Applied Biosystems). Gene expression was quantified relative to the expression of the housekeeping gene (GAPDH), normalized to control by standard 2^-ΔΔCT^ calculation. The primers used in the real-time quantitative PCR were as follows: CCL2, Forward-CAGCCAGATGCAATCAATGCC and reverse-TGGAATCCTGAACCCACTTCT; CXCL10, Forward-GTGGCATTCAAGGAGTACCTC and reverse-TGATGGCCTTCGATTCTGGATT; GAPDH, Forward-GCACCGTCAAGGCTGAGAAC and reverse-TGGTGAAGACGCCAGTGGA. The primers used for real-time quantitative PCR analysis synthetized by Sangon Biotech. Liver biopsy samples were handled in BSL-2 (Biosafety Level 2) laboratory using standard precautions. The steps of Real-Time Quantitative PCR are the same to MΦ-THP-1 cells, except homogenized in liquid nitrogen at the outset.

### Western Blotting

MΦ-THP-1 cells were plated at a density of 2 × 10^6^ cell/well in a 6-well plate. MΦ-THP-1 were pretreated with specific inhibitor in different doses for 30 min and then treated with HCV core protein (10 μg/mL) for 30 min. MΦ-THP-1 cells were lysed with RIPA Lysis Buffer (Beyotime) supplemented with 1 mM phenylmethylsulfonyl fluoride (PMSF, BioSharp) and phosphate inhibitors (Solarbio). The protein concentration was determined by BCA protein Assay Kit (Solarbio). Equal amount of protein was loaded on SDS PAGE gels and transferred onto the PVDF membrane (Millipore), then blocked with 5% nonfat milk in 1 × TBST for 1 h followed by incubation with specific primary antibody overnight at 4°C. The membrane was incubated with HRP-conjugated secondary antibody for 1 h at room temperature. After washing, the membranes were developed with Western Lightning plus-ECL reagent (PerkinElmer) and detected with a Synoptics Syngene Bioimaging instrument (Synoptics). GAPDH was used as control for immunoblotting.

### Co-Immunoprecipitation

HEK 293T cells were plated at a density of 1.5 × 10^6^ cell/well in a 6cm dish. and co-transfected with SFB-Flag-gC1qR and pcDNA3.1-HCV core plasmid (co-transfected with SFB-Flag-gC1qR and pcDNA3.1 as control group). 48 h after transfection, cells were lysed with NTEN buffer (20mM Tris-HCl, pH 8.0, 100mM NaCl, 1 mM EDTA, 0.5% Nonidet P-40) containing 1 mM phenylmethylsulfonyl fluoride (PMSF, BioSharp) and phosphate inhibitors (Solarbio) on ice for 30 minutes. Clear cell lysates were incubated with Streptavidin Agarose (Sigma S1638) overnight at 4°C. Beads were then washed 4 times with NTEN buffer and boiled in 2 × loading buffer. Proteins were separated by SDS-PAGE. PVDF membranes were blocked in 5% nonfat milk in 1 × TBST buffer and then probed with antibodies of FLAG and HCV Core protein.

### ELISA Assay

Macrophages were treated with HCV core protein (10 μg/mL) for 24 h. Cell culture supernatants were collected and stored at -80°C in refrigerator. The amounts of CCL2 and CXCL10 present in culture supernatants were determined using specific Human CCL2 (ELH-MCP-1, Raybiotech) and Human CXCL10 (ELH-IP10, Raybiotech), Mouse CCL2 (EK287, MultiSciences) and Mouse CXCL10 (EMC121, NeobioScience) ELISA kit. The ELISA experiments were performed according to the manufacturer’s protocol.

### Dual Luciferase Reporter Assay

HEK 293T cells were plated at a density of 3.5 × 10^4^ cells/well in 96-well plates. and co-transfected with 0.3 μg of NF-κB p65 expressing plasmid (pmCherry-HA-p65), 0.2 μg of promoter reporter plasmid (pGL4.10-CCL2/pGL4.10-CXCL10) and 0.02 μg of the pRL-TK plasmid (co-transfected with promoter reporter plasmid and pRL-TK plasmid as control group). 48 h after transfection, Firefly Luciferase and Renilla Luciferase activities were detected with the Dual Glo luciferase assay system (RG088S, Beyotime) according to the manufacturer’s instructions. The luminescence was measured with BioTek Synergy HT2 multiscan spectrum.

### Statistical Analysis

Data from three independent experiments were expressed as the mean ± standard deviation (SD). SPSS version 16.0 software was used for statistical analyses. The comparisons were performed by using Student’s t test or one-way analysis of variance (ANOVA). *p*<0.05 was considered to indicate a statistically significant difference.

## Results

### Identification of Differentially Expressed Genes in Macrophages Treated With HCV Core Protein

**Figure 1:**
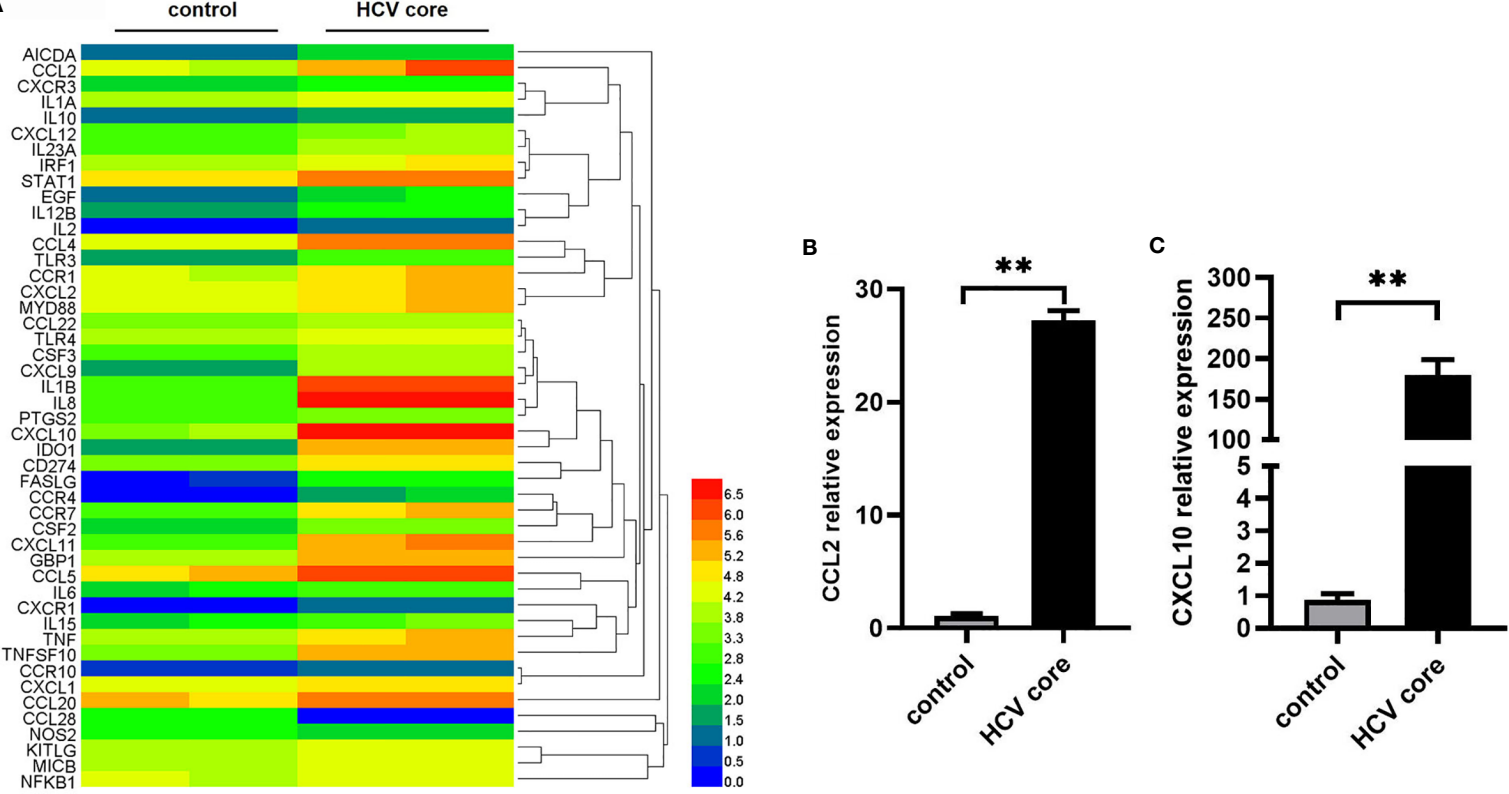
Identification of differentially expressed genes in macrophages treated with HCV core protein. **(A)** Heat map showing the gene expression profile data in macrophages (MΦ-THP-1) treated or untreated with HCV core protein (10 μg/mL) for 8 h based on PCR array analysis. Red: up-regulation; Blue: down-regulation. **(B, C)** MΦ-THP-1 were treated or untreated with HCV core protein (10 μg/mL) for 8 h. Relative expressions of CCL2 and CXCL10 were determined by quantitative real-time PCR (qPCR). Data are shown as mean ± SD for three independent experiments. **p < 0.01.

To explore the effect of HCV core protein on macrophages, we examined the inflammation-associated gene expression in MΦ-THP-1 after treatment with HCV core protein (10 μg/mL) for 8 h while MΦ-THP-1 treated with PBS was taken as control group. PCR array analysis was performed by Shanghai Kangcheng biological company. We observed that 47 genes are differentially expressed in HCV core protein treated samples compared with control samples, among which 45 genes were significantly up-regulated and 2 genes were significantly down-regulated. The expression profiling of these genes is shown by a heat map (**Figure 1A**). In addition, the detailed information for significantly altered genes, including gene names, gene locations and gene functions have been shown in **Supplementary Table 1**. As chemokines are critical for regulating the progression of inflammation disease (43), we focused on two chemokines CCL2 and CXCL10 which upregulated significantly. To confirm the results of the chip, we detected the expression of CCL2 and CXCL10 in MΦ-THP-1 cells using Real-Time Quantitative PCR. The results obtained were consistent with the chip results, CCL2 mRNA level was up-regulated by 27 folds in the presence of HCV core protein (**Figure 1B**), and CXCL10 was up-regulated by 179 folds (**Figure 1C**).

### HCV Core Protein Up-Regulates CCL2 and CXCL10 Expression in Macrophages

**Figure 2:**
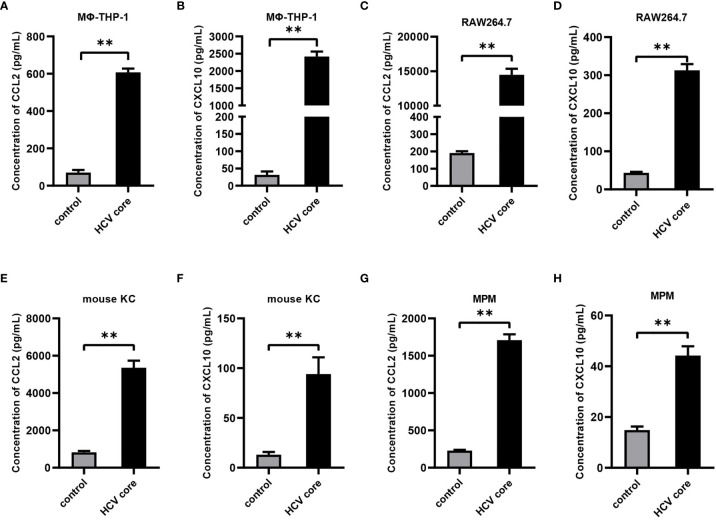
HCV core protein up-regulates CCL2 and CXCL10 expression in macrophages. Human macrophages cell line (MΦ-THP-1) **(A, B)**, mouse macrophage cell line RAW 264.7 **(C, D)**, mouse Kupffer cells (KC) **(E, F)** and mouse peritoneal macrophages (MPM) **(G, H)** were treated or untreated with HCV core protein (10 μg/mL) for 24 h. Cell culture supernatants were collected and analyzed for CCL2 and CXCL10 production using ELISA kits. Data are shown as mean ± SD for three independent experiments. **p < 0.01.

To evaluate the effect of HCV core protein on CCL2 and CXCL10 expression, human macrophage cell line (MΦ-THP-1 cells), mouse macrophage cell line RAW 264.7, mouse Kupffer cells (KC) and mouse peritoneal macrophages (MPM) were treated with HCV core protein for 24 h and protein levels of CCL2 and CXCL10 in the supernatants were determined by ELISA. As shown in **Figures 2A–H**, the expression of chemokines CCL2 and CXCL10 protein were significantly increased in the HCV core protein treated group relative to control group. These data suggested that CCL2 and CXCL10 are elevated in human and mouse macrophages treated by HCV core protein and may play an important role in HCV infection. However, normal liver cell line (L02) treated with HCV core protein did not show upregulation of CCL2 and CXCL10 (data not shown) which ruling out its possible role in CCL2 and CXCL10 expression.

### gC1qR Is Involved in the Expression of CCL2 and CXCL10 in Macrophages

**Figure 3:**
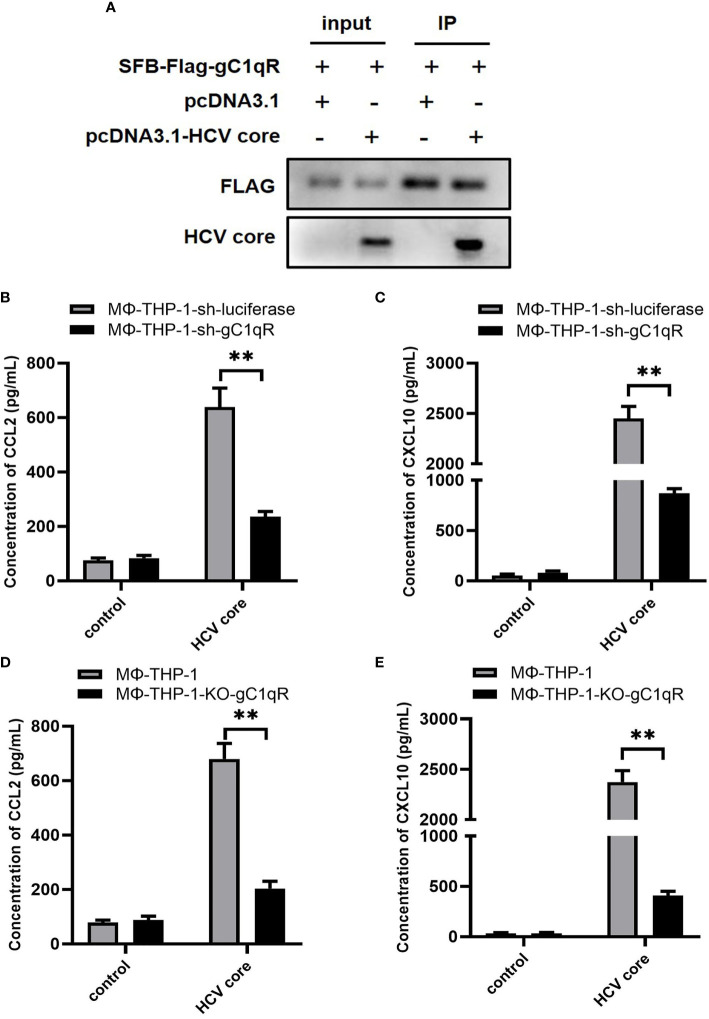
gC1qR is involved in the expression of CCL2 and CXCL10 in macrophages. **(A)** HEK 293T cells were co-transfected with SFB-Flag-gC1qR and pcDNA3.1-HCV core plasmid (co-transfected SFB-Flag-gC1qR and pcDNA3.1 plasmid as control group) for 48 h. Cells were lysed with NTEN buffer and analyzed by immunoprecipitation and immunoblotting using antibodies of FLAG and HCV Core protein. **(B, C)** gC1qR knock-down cell line (MΦ-THP-1-sh-gC1qR) and control cell line (MΦ-THP-1-sh-luciferase) were either treated or untreated with HCV core protein (10 μg/mL) for 24 h. Cell culture supernatants were collected and analyzed for CCL2 and CXCL10 production using ELISA kits. **(D, E)** gC1qR knock-out cell line (MΦ-THP-1-KO-gC1qR) and control cell line (MΦ-THP-1) were either treated or untreated with HCV core protein (10 μg/mL) for 24 h. Cell culture supernatants were collected and analyzed for CCL2 and CXCL10 production using ELISA kits. Data are shown as mean ± SD for three independent experiments. **p < 0.01.

In previous study, we have shown that HCV core protein interacts with gC1qR by pull-down assay (36). Here, we validated this finding by transfecting HEK 293T cells with plasmids expressing gC1qR and HCV core followed by co-immunoprecipitation assay. As shown in **Figure 3A**, gC1qR could combine with HCV core protein. Next, to investigate the role of gC1qR in CCL2 and CXCL10 expression, we assessed the expression of CCL2 and CXCL10 in the supernatant of gC1qR knock-down cell line (MΦ-THP-1-sh-gC1qR) and gC1qR knock-out cell line (MΦ-THP-1-KO-gC1qR) treated with HCV core protein. As shown in **Figures 3B–E**, the protein level of CCL2 and CXCL10 in MΦ-THP-1-sh-gC1qR cells and MΦ-THP-1-KO-gC1qR cells were significantly reduced compared to control group. However, CCL2 and CXCL10 expression were not completely abolished even in gC1qR knock-out cells. These results indicate that gC1qR serves as an important receptor for HCV core protein to induce CCL2 and CXCL10 expression in macrophages.

### NF-κB Signaling Pathway Plays Pivotal Role in CCL2 and CXCL10 Expression in Macrophages Induced by HCV Core Protein

**Figure 4:**
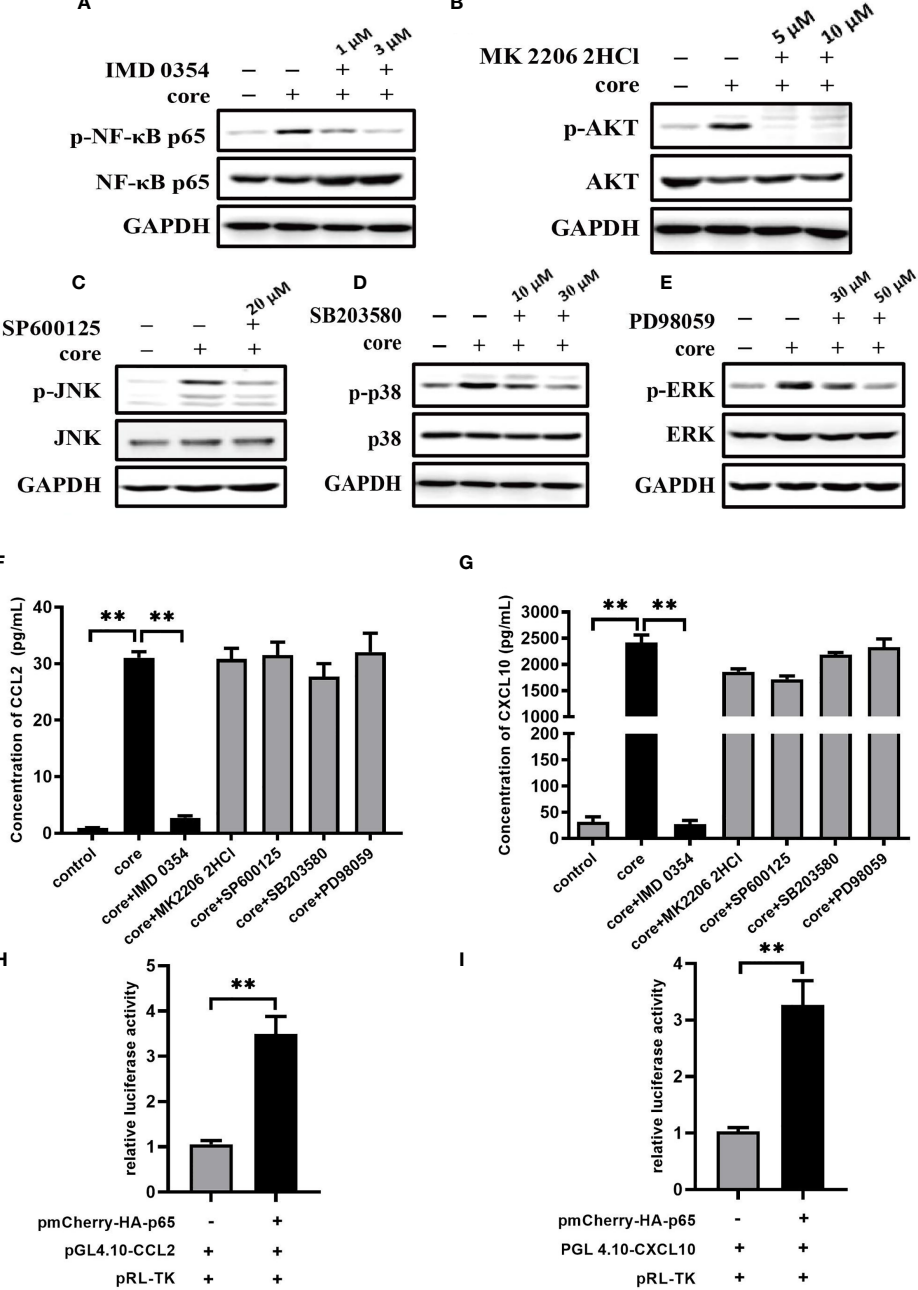
NF-κB signaling pathway plays pivotal role in CCL2 and CXCL10 expression in macrophages induced by HCV core protein. **(A–E)** MΦ-THP-1 were pretreated with specific inhibitor in different doses for 30 min and then treated with HCV core protein for 30 min. Cells were lysed and analyzed by immunoblotting with anti-p-NF-κB p65, anti-NF-κB p65, anti-p-AKT, anti-AKT, anti-p-JNK, anti-JNK, anti-p-p38, anti-p38, anti-p-ERK, anti-ERK and anti-GAPDH antibodies to determine the optimum concentration of inhibitors: IKKβ inhibitor IMD 0354 was 3 μM, Akt inhibitor MK 2206 2HCl was 5 μM, JNK inhibitor SP600125 was 20 μM, P38 inhibitor SB203580 was 30 μM, ERK inhibitor PD98059 was 50 μM. **(F, G)** MΦ-THP-1 were pretreated with optimal concentration of specific inhibitor and then treated with HCV core protein for 24 h. The supernatants were analyzed by the ELISA to detect CCL2 and CXCL10 production. **(H, I)** Luciferase activities were detected in HEK 293T cells co-transfected with NF-κB p65expressing plasmid, CCL2 or CXCL10 reporter plasmid and pRL-TK plasmid as described in Materials and Methods (co-transfected with promoter reporter plasmid and pRL-TK plasmid as control group). Data are presented as the relative ratio of Firefly luciferase activity to Renilla luciferase activity. Data are shown as mean ± SD for three independent experiments. **p < 0.01.

From our previous study, we have demonstrated that HCV core/gC1qR engagement triggers the activation of NF-κB pathway, PI3K/AKT pathway and JNK, P38, ERK pathways (36). To explore the mechanism of CCL2 and CXCL10 expression, we performed specific inhibitors to assess the effect of above signaling pathways on CCL2 and CXCL10 expression. Firstly, we determined the optimal concentration for inhibitors: IMD 0354 (inhibit IKKβ to block NF-κB pathway), MK 2206 2HCl (inhibit AKT to block PI3K/AKT pathway), SP600125 (inhibit JNK to block JNK pathway), SB203580 (inhibit p38 to block p38 pathway), PD98059 (inhibit ERK to block ERK pathway). As shown in **Figures 4A–E**, the optimal concentration of IKKβ inhibitor IMD 0354 was at 3 μM, Akt inhibitor MK 2206 2HCl was 5 μM, JNK inhibitor SP600125 was 20 μM, P38 inhibitor SB203580 was 30 μM, ERK inhibitor PD98059 was 50 μM. Secondly, to explore the effect of inhibitors on CCL2 and CXCL10 expression, we measured the protein level of CCL2 and CXCL10 in the supernatant of MΦ-THP-1 pretreated with specific inhibitor for 30 min and treated with HCV core protein for 24 h. As shown in **Figures 4F, G**, blocking of NF-κB pathway significantly reduces the expression of CCL2 or CXCL10, while PI3K/AKT, JNK, P38, ERK pathway inhibitors did not show any effects. These results suggest that NF-κB pathway plays important role in CCL2 and CXCL10 expression in macrophages treated with HCV core protein.

Further, we investigated the role of NF-κB on transcriptional regulation of CCL2 and CXCL10 by Dual Luciferase Reporter Assay through co-transfecting HEK 293T cells with NF-κB p65 expressing plasmid (pmCherry-HA-p65), CCL2 or CXCL10 reporter plasmid (pGL4.10-CCL2/pGL4.10-CXCL10) and pRL-TK plasmid. Results from luciferase activity assays showed that transcriptional activity of CCL2 or CXCL10 were higher in the presence of NF-κB p65 protein (**Figures 4H, I** **)**. The results suggested that NF-κB p65 protein can regulate the transcriptional level of CCL2 or CXCL10.

### CCL2 and CXCL10 Expression Significantly Increased in Liver Biopsy of HCV Patients

**Figure 5:**
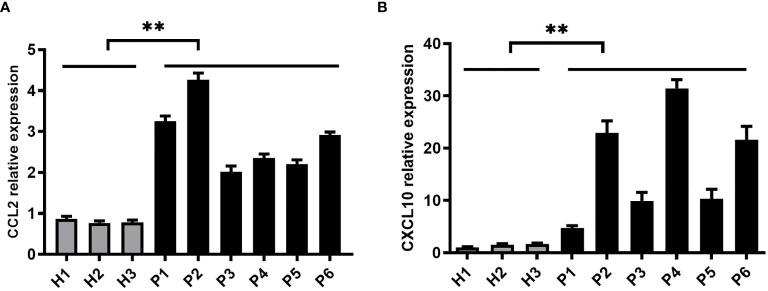
CCL2 and CXCL10 expression is significantly increased in liver biopsy samples of HCV patients. **(A, B)** Six liver biopsy samples from patients infected with HCV and three control samples from healthy liver tissues were collected. The samples were handled in BSL-2 laboratory using standard precautions. Relative expressions of CCL2 and CXCL10 were determined by quantitative real-time PCR (qPCR). Data are shown as mean ± SD for three independent experiments. **p < 0.01.

To validate the results obtained *in vitro*, we collected liver biopsy samples from patients infected with HCV from the Third Affiliated Hospital of Hebei Medical University while healthy liver tissues were taken as control group. HCV liver biopsy samples were handled in BSL-2 (Biosafety Level 2) laboratory using standard precautions. These samples were assessed for CCL2 and CXCL10 expression levels by real-time PCR assay. We detected higher expression of CCL2 and CXCL10 in HCV liver biopsy samples than the control group (**Figures 5A, B** **)**. These results indicate that HCV infection significantly increases the expression of CCL2 and CXCL10 to enhance HCV mediated inflammatory responses.

## Discussion

It is well known that sustained inflammation is a major cause of liver damage during chronic HCV infection (44). HCV infection tends to develop into chronicity through altered or exacerbated signaling of pro-inflammatory cytokines and chemokines which may compromise the viral clearance and promote the development of liver fibrosis and hepatocellular carcinoma (45–49). However, the mechanism of HCV chronic inflammation remains unclear.

Many literatures suggested that HCV core protein exhibits multiple functions to affect host immunity by interacting with immunocytes. For example, the interaction of HCV core protein with gC1qR has been shown to inhibit the proliferation of T lymphocytes by reducing IL-2 and IL-2Rα gene transcription (50) and impair IL-12 secretion by macrophages through induction of negative regulators such as Tim-3, PD-1 and SOCS-1 (51, 52). In addition, HCV core protein could induce TLR tolerance in macrophages through its interaction with TLR2 (53). Our previous study showed that HCV core protein combined with gC1qR could induce A20 expression and promote chronic inflammation. In this study, we confirmed the interaction between HCV core protein and gC1qR by co-immunoprecipitation. Further, knock-down or knock-out gC1qR in macrophages significantly reduced the production of CCL2 and CXCL10 when treated with HCV core protein indicating that gC1qR play a key role in CCL2 and CXCL10 expression. However, knock-out of gC1qR in macrophages did not completely abolish CCL2 and CXCL10 expression so we speculated that other receptors may be also involved in it. Therefore, our data indicate that gC1qR serves as one of the receptors for HCV core protein to induce CCL2 and CXCL10 expression in macrophages. Whether other receptors in addition to gC1qR are required for CCL2 and CXCL10 expression and, if any, how these receptors are involved in HCV infection remains to be determined.

Chemokines are a family of small proteins (8–12kDa) which regulate migration of immunocytes by binding to their receptors (54–56). CCL2 (Monocyte chemoattractant protein-1, MCP-1) is an important chemokine involved in the recruitment of monocytes/macrophages and T cells to areas of inflammation *via* CCR2. CXCL10 (interferon-inducible protein-10, IP-10) binding to its receptor CXCR3 attracts CXCR3^+^ cells such T lymphocytes, monocytes and NK cells (57, 58). Saraiva et al. have reported that serum levels of CCL2 were increased in HCV-infected patients compared to control group (59). Nguyen et al. have shown that high concentrations of CXCL10 and CCL2 were associated with a poor anti-HCV response (60). Other studies have shown that HCV can induce CXCL10 expression in hepatocytes which take part in the pathogenesis of chronic HCV infection (49, 61). In case of chronic HCV infection, CXCL10 can be considered as a marker of liver fibrosis (62–64). All the above studies suggested that high expression of chemokines CCL2 and CXCL10 in patients with HCV infection is related to disease progression and poor prognosis. In this study, to further verify the effect of HCV core protein on CCL2 or CXCL10 expression, we isolated Kupffer cells from the liver of the mice. Results obtained from mice liver Kupffer cells were consistent with those obtained from the cell lines of macrophages (MΦ-THP-1 and RAW 264.7 cells), CCL2 and CXCL10 were elevated when treated with HCV core protein, the same results were also obtained in mouse peritoneal macrophages. In addition, we observed that the expression of CCL2 and CXCL10 in liver biopsy of HCV infected patients were significantly higher than those in healthy individual group. However, we did not observe up-regulation of CCL2 and CXCL10 in normal liver cell line (L02) in the presence or absence of HCV core protein (data not shown). In brief, our results suggest that it is macrophages treated with HCV core protein increase the expression of CCL2 and CXCL10.

To explore the mechanism of CCL2 and CXCL10 expression in macrophages, we investigated NF-κB pathway, PI3K/AKT pathway and JNK, P38, ERK pathways which could be activated by HCV core protein. We employed specific inhibitors to inhibit specific signaling pathways and found that CCL2 and CXCL10 protein levels were significantly inhibited after blocking NF-κB pathway, while PI3K/AKT, JNK, P38, ERK pathway inhibitors did not show any effects. These data suggest that NF-κB pathway plays a major role in CCL2 and CXCL10 expression. NF-κB plays crucial role in host response to infection and functions as a dimeric protein with different members of the Rel family proteins (p50/NF-κB1, p52/NF-κB2, c-Rel/Rel, p65/Rel-A and Rel-B) (65, 66). In this study, we examined the role of NF-κB p65 in the transcriptional regulation of CCL2 and CXCL10. The data from Dual Luciferase Reporter Assay suggested that NF-κB p65 could regulate the transcriptional level of CCL2 and CXCL10.

In summary, the data reported here demonstrates that HCV core protein upregulates the expression of CCL2 and CXCL10 by interacting with gC1qR in macrophages and NF-κB pathway plays pivotal role in CCL2 and CXCL10 expression. Our findings further suggest that targeting the NF-κB pathway and gC1qR can be a potential therapeutic strategy against HCV-related inflammatory damage, which not only blocks CCL2 and CXCL10 generation but also impairs its chemotactic function. These findings may also shed light on the understanding of how leukocytes migrate into the liver and exaggerate host-derived immune responses and may provide novel mechanism in HCV chronic inflammation.

## Data Availability Statement

The datasets presented in this study can be found in online repositories. The names of the repository/repositories and accession number(s) can be found in the article/**Supplementary Material**.

## Ethics Statement

The studies involving human participants were reviewed and approved by Medical Ethics Committee of Hebei Medical University. The patients/participants provided their written informed consent to participate in this study. The animal study was reviewed and approved by Laboratory Animal Ethical and Welfare Committee of Hebei Medical University.

## Author Contributions

XS designed experiments and wrote the manuscript. XG, YZ, SY, ML and XS performed the experiments. YW provided patients samples. RR analyzed data. ZY prepared the figures and tables. LW revised and edited the manuscript. All authors contributed to the article and approved the submitted version.

## Funding

This work was supported by grants from the National Science Foundation of China (81801560, 81702827 and 81802014), the Science and Technology Planning Project of Hebei Province (H2019206614) and the Science and Technology Research Projects of the Colleges and Universities of Hebei Province (ZD2021071, QN2017105).

## Conflict of Interest

The authors declare that the research was conducted in the absence of any commercial or financial relationships that could be construed as a potential conflict of interest.

## Publisher’s Note

All claims expressed in this article are solely those of the authors and do not necessarily represent those of their affiliated organizations, or those of the publisher, the editors and the reviewers. Any product that may be evaluated in this article, or claim that may be made by its manufacturer, is not guaranteed or endorsed by the publisher.
