# Supplementary material for: HCV Core Protein Induces Chemokine CCL2 and CXCL10 Expression Through NF-κB Signaling Pathway in Macrophages
Source: Front Immunol. 2021 Aug 31;12:654998. doi: 10.3389/fimmu.2021.654998 (PMC8438213; doi:10.3389/fimmu.2021.654998)
Supplement: Supplementary file 1 [file DataSheet_1.pdf]

Supplementary Table 1 Detailed information for genes that have been significantly altered in MΦ-THP-1 treated with HCV core protein based on PCR Array Analysis.

Gene	Location	Function
Upregulated genes		
**CCL2**	**17q11.2-q21.1**	**Chemotactic factor that attracts monocytes and basophils. Augments monocyte anti-tumor activity.**
CCL20	2q36.3	Chemotactic factor that attracts lymphocytes . Inhibits proliferation of myeloid progenitors in colony formation assays.
CCL22	16q13	Chemotactic for monocytes, dendritic cells and natural killer cells.
CCL4	17q21-q23	Monokine with inflammatory and chemokinetic properties. Binds to CCR5.
CCL5	17q11.2-q12	Chemoattractant for blood monocytes, memory T-helper cells and eosinophils. Binds to CCR1, CCR3, CCR4 and CCR5.
CCR1	3p21	Receptor for a C-C type chemokine. Binds to MIP-1-alpha, MIP-1-delta, RANTES, and MCP-3.
CCR7	17q12-q21.2	Receptor for the MIP-3-beta chemokine.
CD274	9p24.1	Costimulatory signal, essential for T-cell proliferation and production of IL10 and IFNG.
CSF3	17q11.2-q12	Induce granulocytes.
**CXCL10**	**4q21**	**Chemotactic for monocytes and T-lymphocytes. Binds to CXCR3**
CXCL12	10q11.1	Chemoattractant active on T-lymphocytes, monocytes, but not neutrophils.
CXCL9	4q21	Cytokine that affects the growth, movement, or activation state of cells that participate in immune and inflammatory response. Chemotactic for activated T-cells. Binds to CXCR3.
CXCR3	Xq13	Receptor for the C-X-C chemokine CXCL9, CXCL10 and CXCL11.Bind to CCL21.
EGF	4q25	Stimulate the growth of various epidermal and epithelial tissues
FASLG	1q23	Cytokine that binds to TNFRSF6/FAS, a receptor that transduces the apoptotic signal into cells.
GBP1	1p22.2	Hydrolyzes GTP to GMP in two consecutive cleavage reactions. Exhibits antiviral activity against influenza virus.
IDO1	8p12-p11	Catalyzes the cleavage of the pyrrol ring of tryptophan and incorporates both atoms of a molecule of oxygen
IL10	1q31-q32	Inhibits the synthesis of a number of cytokines, including IFN-gamma, IL-2, IL-3,

		TNF and GM-CSF produced by activated macrophages and by helper T-cells.
IL12B	5q31.1-q33.1	Cytokine that can act as a growth factor for activated T and NK cells, enhance the lytic activity of NK/lymphokine-activated killer cells, and stimulate the production of IFN-gamma by resting PBMC
IL15	4q31	Cytokine that stimulates the proliferation of T-lymphocytes.
IL1B	2q14	IL-1 stimulates thymocyte proliferation by inducing IL-2 release, B-cell maturation and proliferation, and fibroblast growth factor activity.
IL23A	12q13.13	Associates with IL12B to form the IL-23 interleukin, a heterodimeric cytokine which functions in innate and adaptive immunity.
IL6	7p21-p15	Cytokine with a wide variety of biological functions
IL8	4q13-q21	IL-8 is a chemotactic factor that attracts neutrophils, basophils, and T-cells, but not monocytes. It is also involved in neutrophil activation.
IRF1	5q23-q31	Transcriptional regulator which displays a remarkable functional diversity in the regulation of cellular responses.
KITLG	12q22	Ligand for the receptor-type protein-tyrosine kinase KIT.
MICB	6p21.3	Acts as a stress-induced self-antigen that is recognized by gamma delta T cells. Ligand for the KLRK1/NKG2D receptor. Binding to KLRK1 leads to cell lysis.
MYD88	3p22	Adapter protein involved in the Toll-like receptor and IL-1 receptor signaling pathway in the innate immune response.
STAT1	2q32.2-q32.3	Signal transducer and transcription activator that mediates cellular responses to interferons (IFNs), cytokine KITLG/SCF and other cytokines and other growth factors.
TLR3	4q35	TLR3 is a nucleotide-sensing TLR which is activated by double-stranded RNA, a sign of viral infection.
TLR4	9q33.1	Cooperates with LY96 and CD14 to mediate the innate immune response to bacterial lipopolysaccharide(LPS).
TNF	6p21.3	Cytokine that binds to TNFRSF1A/TNFR1 and TNFRSF1B/TNFBR.
TNFSF10	3q26	Induces apoptosis.
Downregulated gene		
CCL28	5p12	Chemotactic activity for resting CD4, CD8 T-cells and eosinophils. Binds to CCR3 and CCR10 and induces calcium mobilization in a dose-dependent manner.
NOS2	17q11.2-q12	Produces nitric oxide (NO) which is a messenger molecule with diverse functions throughout the body.

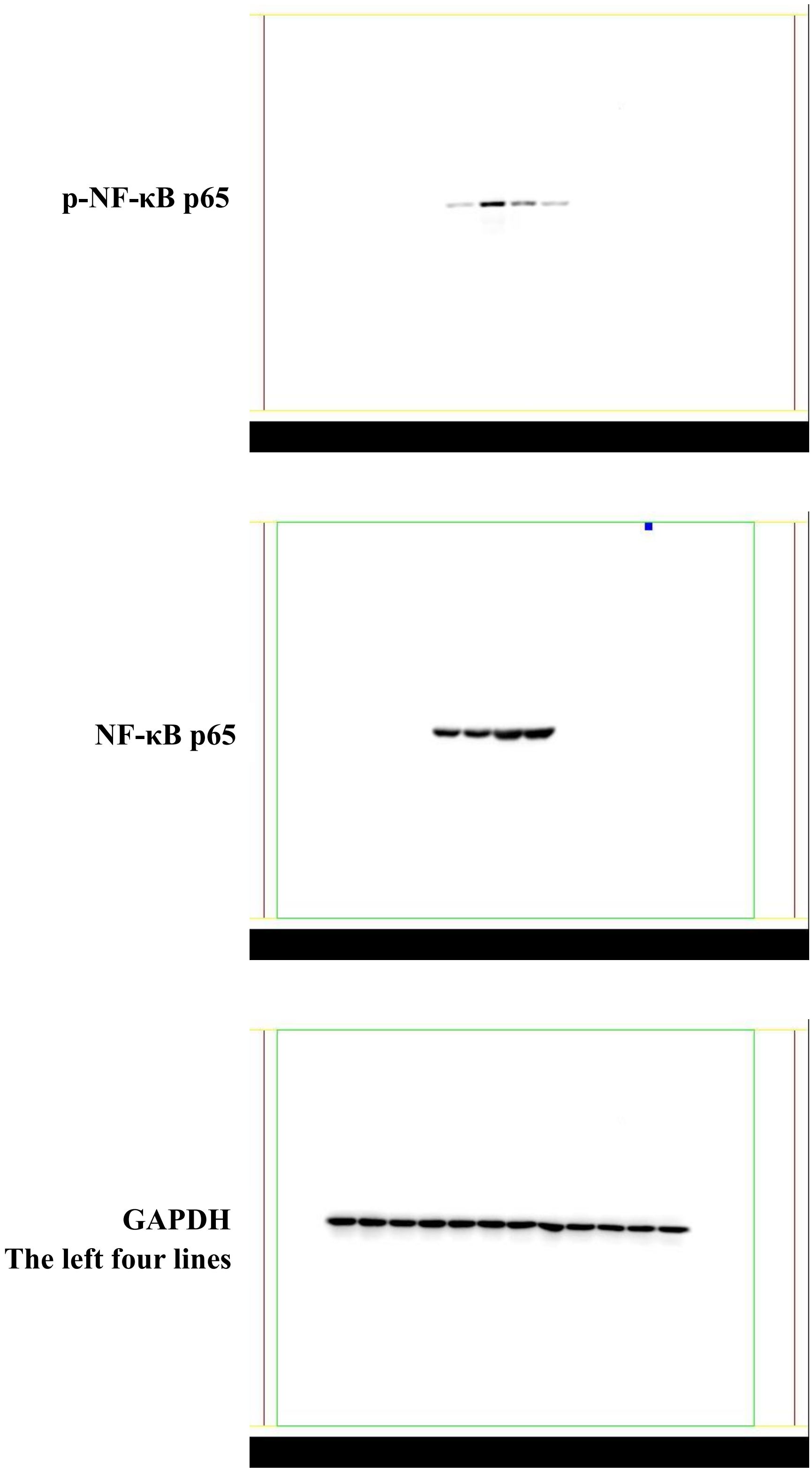

Supplementary Figure 1 MΦ-THP-1 was pretreated with inhibitor IMD 0354(1μM or 3μM) for 30 min and then treated with HCV core protein for 30 min followed by analysis of p-NF-κB p65, NF-κB p65 and GAPDH expression by Western blotting.

p-AKT

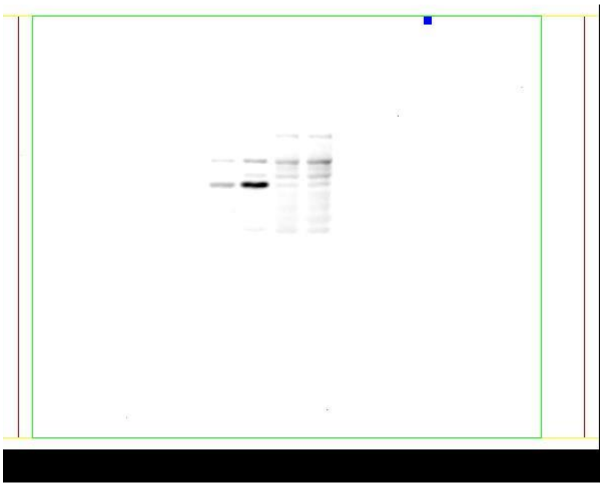

AKT

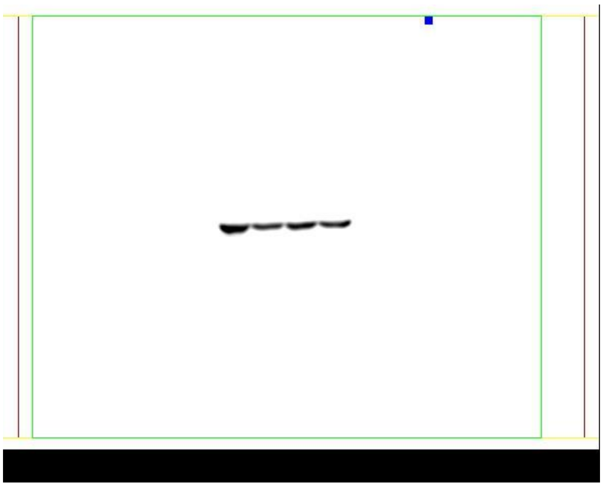

GAPDH

The middle four lines

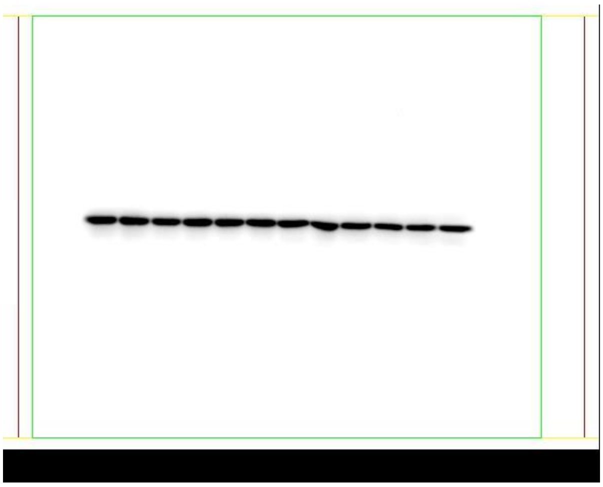

Supplementary Figure 2 MΦ-THP-1 was pretreated with inhibitor MK 2206 2HCl (5 µM or 10 µM) for 30 min and then treated with HCV core protein for 30 min followed by analysis of p-AKT, AKT and GAPDH expression by Western blotting.

p-JNK

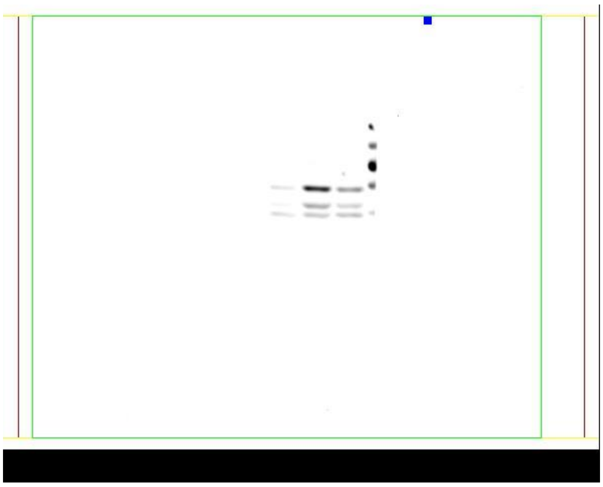

JNK

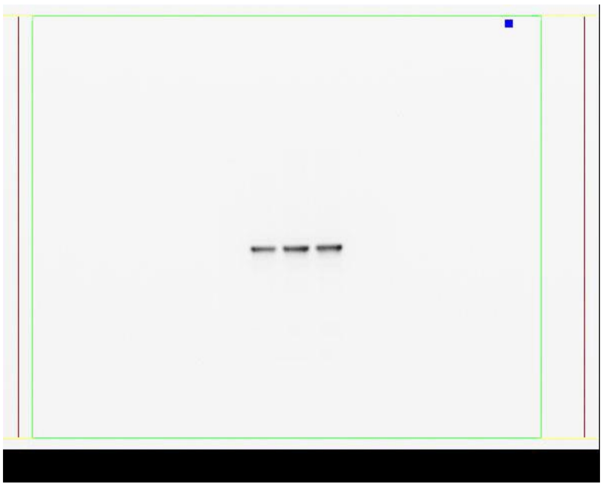

GAPDH
The right three lines

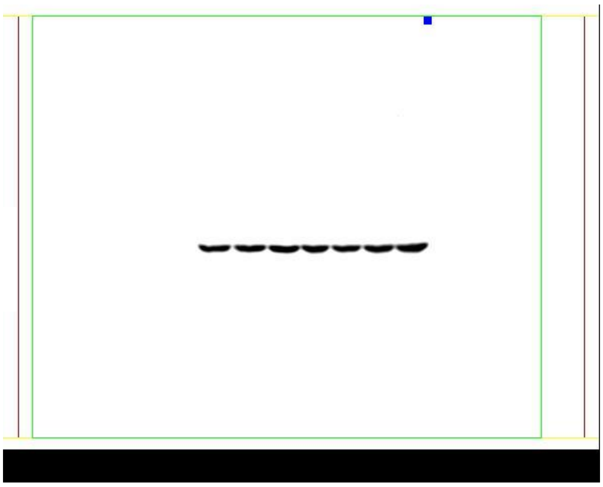

Supplementary Figure 3 MΦ-THP-1 was pretreated with inhibitor SP600125(20 μM) for 30 min and then treated with HCV core protein for 30 min followed by analysis of p-JNK， JNK and GAPDH expression by Western blotting.

p-38

P38

GAPDH

The right four lines

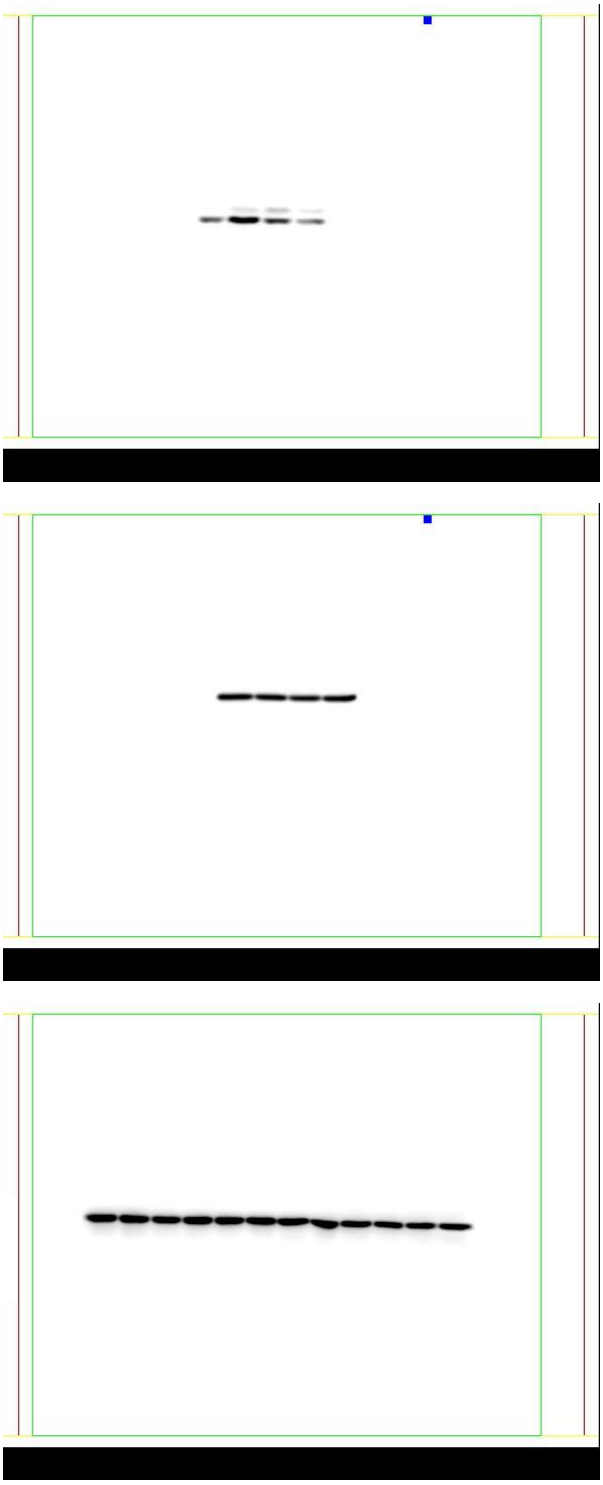

Supplementary Figure 4 MΦ-THP-1 were pretreated with inhibitor SB203580(10 μM or 30 μM) for 30 min and then treated with HCV core protein for 30 min followed by analysis of p-p38, p38 and GAPDH expression by Western blotting.

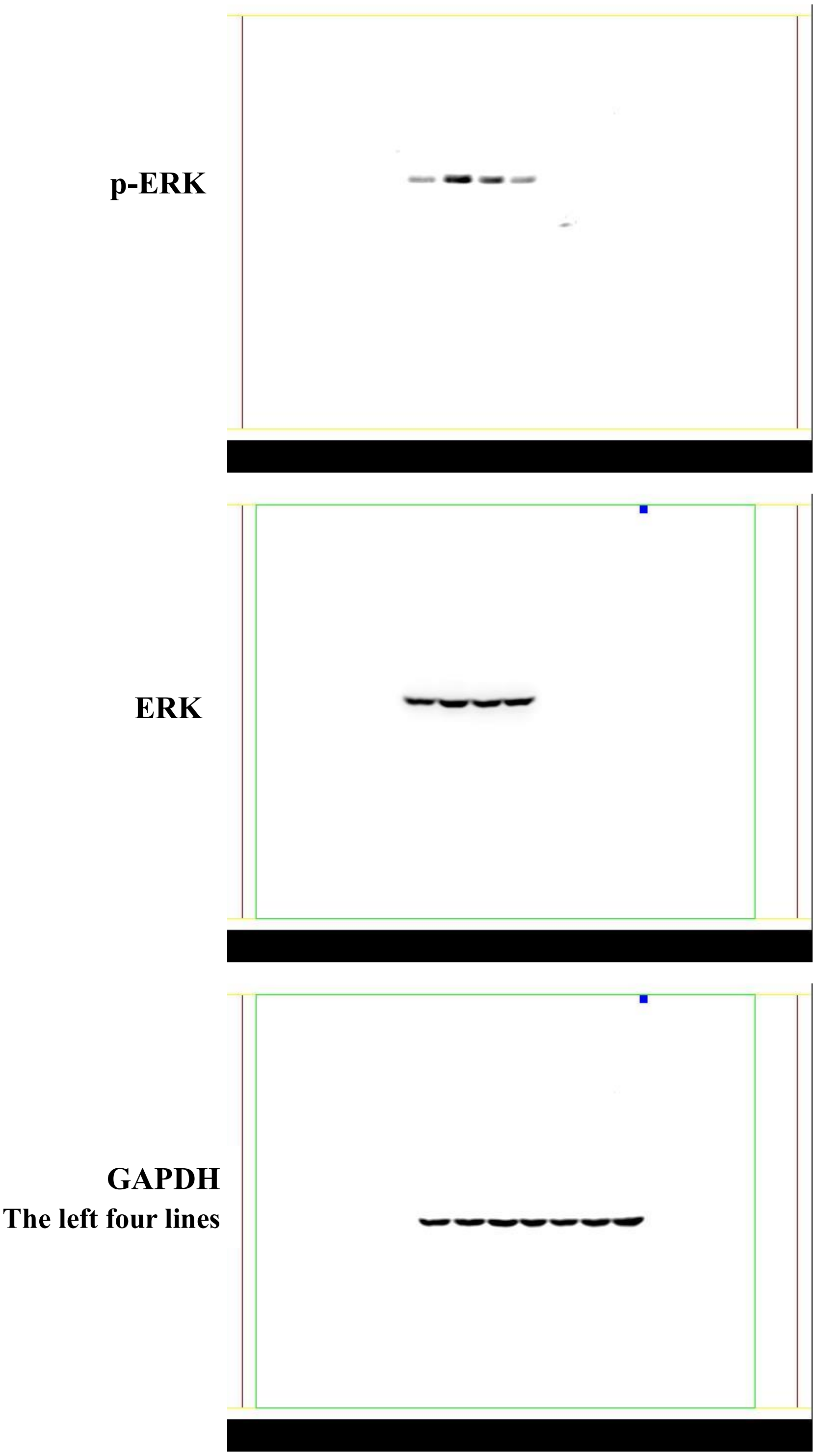

Supplementary Figure 5 MΦ-THP-1 were pretreated with inhibitor PD98059(30μM or 50μM) for 30 min and then treated with HCV core protein for 30 min followed by analysis of p-ERK, ERK and GAPDH expression by Western blotting.

FLAG

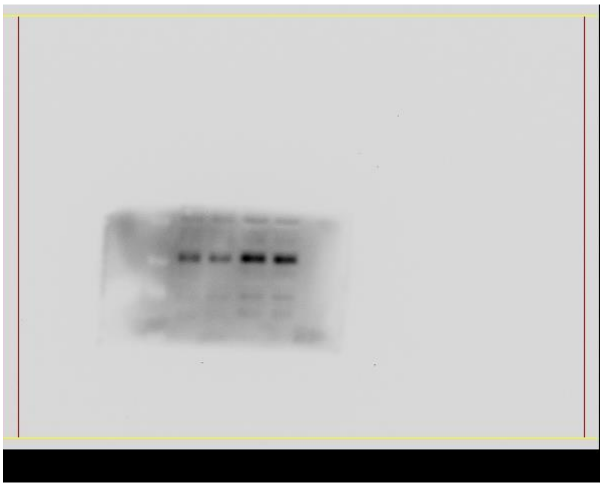

HCV core

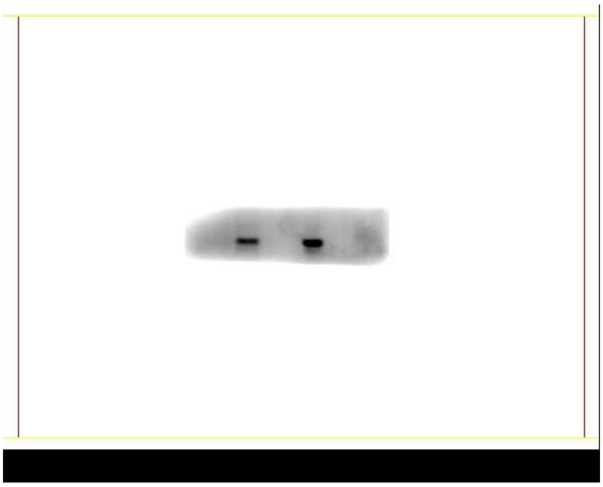

Supplementary Figure 6 HEK 293T cells were co-transfected with SFB-Flag-gC1qR and pcDNA3.1-HCV core plasmids (co-transfected with SFB-Flag-gC1qR and pcDNA3.1 plasmid as control group). 48 h after transfection, cells were lysed with NTEN buffer and analyzed by immunoprecipitation and immunoblotting using the indicated antibodies.
